# Chest X-ray at Emergency Admission and Potential Association with Barotrauma in Mechanically Ventilated Patients: Experience from the Italian Core of the First Pandemic Peak

**DOI:** 10.3390/tomography9060171

**Published:** 2023-12-08

**Authors:** Pietro Andrea Bonaffini, Francesco Stanco, Ludovico Dulcetta, Giancarla Poli, Paolo Brambilla, Paolo Marra, Clarissa Valle, Ferdinando Luca Lorini, Mirko Mazzoleni, Beatrice Sonzogni, Fabio Previdi, Sandro Sironi

**Affiliations:** 1 Department of Radiology, ASST Papa Giovanni XXIII, Piazza OMS, 24127 Bergamo, BG, Italy; 2 School of Medicine, University of Milano-Bicocca, Piazza dell’Ateneo Nuovo, 1, 20126 Milan, MI, Italy; 3 Unit of Intensive Care and Anesthesia 2, Papa Giovanni XXIII Hospital, Piazza OMS, 24127 Bergamo, BG, Italy; 4 Department of Management, Information and Production Engineering, University of Bergamo, Via Pasubio, 7/B, 24044 Dalmine, BG, Italy

**Keywords:** COVID-19, chest radiography, Brixia score, mechanical ventilation, barotrauma

## Abstract

Barotrauma occurs in a significant number of patients with COVID-19 interstitial pneumonia undergoing mechanical ventilation. The aim of the current study was to investigate whether the Brixia score (BS) calculated on chest-X-rays acquired at the Emergency Room was associated with barotrauma. We retrospectively evaluated 117 SARS-CoV-2 patients presented to the Emergency Department (ED) and then admitted to the intensive care unit (ICU) for mechanical ventilation between February and April 2020. Subjects were divided into two groups according to the occurrence of barotrauma during their hospitalization. CXRs performed at ED admittance were assessed using the Brixia score. Distribution of barotrauma (pneumomediastinum, pneumothorax, subcutaneous emphysema) was identified in chest CT scans. Thirty-eight subjects (32.5%) developed barotrauma (25 pneumomediastinum, 24 pneumothorax, 24 subcutaneous emphysema). In the barotrauma group we observed higher Brixia score values compared to the non-barotrauma group (mean value 12.18 vs. 9.28), and logistic regression analysis confirmed that Brixia score is associated with the risk of barotrauma. In this work, we also evaluated the relationship between barotrauma and clinical and ventilatory parameters: SOFA score calculated at ICU admittance and number of days of non-invasive ventilation (NIV) prior to intubation emerged as other potential predictors of barotrauma.

## 1. Introduction

Severe acute respiratory syndrome coronavirus 2 (SARS-CoV-2) was identified in late 2019 in China, and then resulted in a global pandemic declared by the World Health Organization on 11 March 2020 [1]. Bergamo (Italy) was one of the global epicenters during the first pandemic wave, with a dramatic increase in the number of patients requiring invasive ventilation between 20 February and 15 April 2020 [2].

Several studies indicate that patients on mechanical ventilation with COVID-19-related pneumonia have a higher rate of barotrauma than patients with acute respiratory distress syndrome without COVID-19 infection [3,4,5,6]. Barotrauma, a form of ventilator-associated lung injury (VALI), refers to the presence of extra-alveolar air in patients on mechanical ventilation [7,8]. Barotrauma results from a bronchial or alveolar rupture with an air leak into the surrounding tissues and spaces, detectable on chest X-ray (CXR) or CT scans. Depending on air collection, different clinical manifestations of barotrauma exist: pneumomediastinum, when air is introduced along the perivascular sheaths into the mediastinum; subcutaneous emphysema, when the mediastinal air is decompressed along cervical fascial planes into the subcutaneous tissues; and pneumothorax, which is an abnormal collection of air in the pleural space [3]. Spontaneous pneumomediastinum or pneumothorax can also occur in non-ventilated SARS-CoV-2 patients, suggesting that COVID-19 predisposes to alveolar rupture in relation to viral and inflammatory injuries, which results in apoptosis and necroptosis of the lung epithelial cells [9,10,11,12,13,14].

Barotrauma can lead to an acute worsening in respiratory function with increased morbidity and mortality, especially in medically compromised patients in Intensive Care Unit (ICU) [15,16]. Nevertheless, no definite radiological models predictive of barotrauma risk have been proposed. Brixia score (BS) is a CXR scoring system designed for COVID-19 pneumonia, combined with demographic, clinical, and laboratory data, that has proved to be a useful tool to predict clinical outcome in COVID-19 patients [17,18].

On these bases, the aims of the present study are (a) to correlate the risk of barotrauma with initial CXR findings in the Emergency Room using BS, and (b) to describe the relationship between barotrauma and clinical and ventilatory parameters derived from ICU and medical records.

## 2. Materials and Methods

The Institutional Review Board (Comitato Etico di Bergamo, Italy) approved this retrospective observational study (COVIDRADPGXXIII01) and waived the written informed consent due to the pandemic contingency.

### 2.1. Study Population

We retrospectively enrolled all COVID-19-proven patients (positive RT-PCR test) presented between 20 February and 15 April 2020 to the Emergency Department (ED) of Papa Giovanni XXIII Hospital in Bergamo, with the following inclusion criteria: (a) CXR performed at ED admission, available for Brixia scoring; (b) available chest CT for barotrauma classification; (c) noninvasive ventilation (NIV) in prior intensive care unit (ICU); and (d) final admission to ICU for mechanical ventilation (MV), with assessment of SOFA (Sequential Organ Failure Assessment) and SAPS (Simplified Acute Physiology Score) II scores. Exclusion criteria were as follows: (a) non-COVID patients, and (b) no available CXR and/or chest CT. Each patient included was then classified into two groups: Group 1, patients with barotrauma during the ICU hospitalization period, and Group 2, patients without barotrauma.

### 2.2. Demographic, Clinical and Laboratory Data Collection

For all included patients, demographic, clinical and laboratory data were collected from patients’ medical records. The recorded data included the following: age, sex, BMI, symptoms, comorbidities, date from admittance, type of barotrauma, days of NIV prior MV, SOFA and SAPS II scores calculated by referring physicians at ICU admittance [19,20], PaO_2_/FiO_2_ ratio (arterial partial pressure of oxygen [PaO_2_]/fraction of inspired oxygen [FiO_2_]), values of the main stages of patient care and positive-end-expiratory pressure (PEEP) values recorded at certain times of invasive and noninvasive ventilation. Mortality rate was also recorded.

### 2.3. Imaging Acquisition and Analysis—CXR and CT

CXR were acquired in the Emergency Room, employing digital radiographic systems (Definium 8000, GE Healthcare, Chicago, IL, USA; FDR AcSelerate, Fujifilm Corporation, Tokyo, Japan), equipped with automatic exposure control and with 120–150 kV tube voltages ranges. Due to critical clinical conditions, the largest number of CXR was acquired in a single antero-posterior (AP) projection with patients in supine or sitting position (n = 86); a smaller amount of CXR (n = 31) were acquired in orthostasis in posteroanterior (PA) and lateral (LAT) projections.

Available AP and PA images were evaluated by a senior radiologist with 20 years of experience (P. B.). CXR were scored using the Brixia scoring (BS) system [17]. BS lungs are divided into six zones on frontal CXR (upper, middle and lower zones) and a score is assigned to each zone from 0 to 3 based on types of pulmonary infiltrates (0, no lung abnormalities; 1, interstitial infiltrates; 2, interstitial and alveolar infiltrates (interstitial predominance); 3, interstitial and alveolar infiltrate (alveolar predominance)) for a maximum value of 18. During the course of the disease, chest CT scans were performed according to clinical requests: such as suspicion or confirmation of barotrauma detected on CXR and/or assessment of barotrauma extent (pneumothorax, pneumomediastinum, subcutaneous emphysema). CTs were acquired either unenhanced or enhanced in case of suspicion of pulmonary embolism in the supine position, and in full inspiration, covering from the lung bases to the apex with either a 64- or a 16-slice scanner (Brilliance 64 and MX 16-slice; Philips Medical Systems, Best, The Netherlands).

### 2.4. Statistical Analysis

Quantitative variables are expressed using mean and standard deviation or as median and interquartile ranges (IQR). These indicators are computed for both continuous and discrete variables with median and IQR being more appropriate for discrete variables.

In order to analyze the effectiveness of BS, the two CXR data sets (barotrauma vs. no barotrauma) have been tested against the null hypothesis that the BS is the same for both using unpaired the Mann–Whitney U test, which is a non-parametric test suitable for hypothesis testing on ordinal data that allows to investigate whether the two populations have the same median value (null hypothesis). If the result of the test is significant and the alternative hypothesis is accepted, then it can be concluded that there is a statistically significant difference between the medians of the two samples. The statistical significance of the two-tailed test is set at 0.05.

Univariate logistic regression has been used to test the effect of several variables on the patient risk of developing barotrauma with a significance level *p* < 0.05. Finally, multivariate logistic regression has been tested to test the joint effect of the most significant variables selected using univariate regression (significance level *p* < 0.05).

## 3. Results

### 3.1. Patient Population

A total of 117 SARS-CoV-2 patients (24 females, 93 males; mean age 59.91) were included in the analysis: 38/117 (32.5%) developed barotrauma (Group 1), 79/117 (67.5%) did not develop barotrauma. In both groups there was an overall male prevalence: 29/38 (76.32%) in Group 1 and 64/79 (81.01%) in Group 2, 2964.

**Figure 1:**
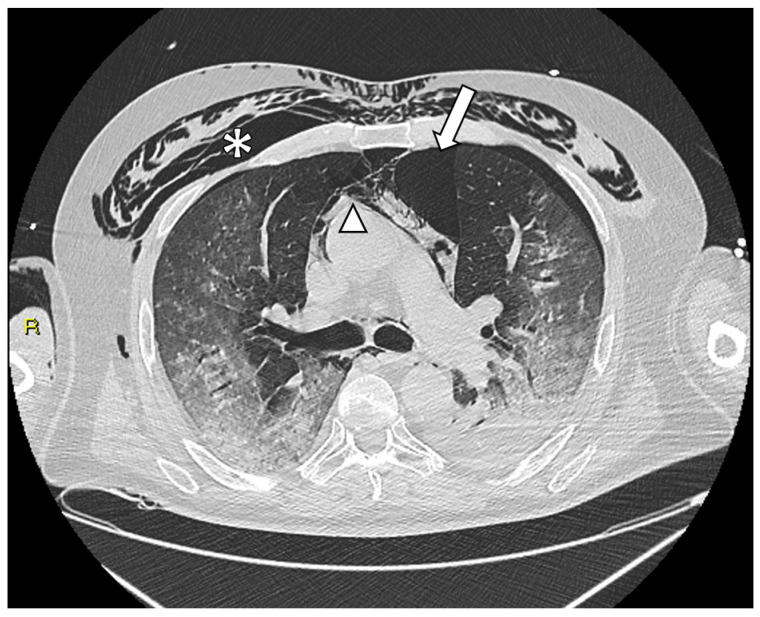
Axial chest CT image of a 62-year-old male with COVID-19 pneumonia (Group 1), who developed pneumothorax (arrow), pneumomediastinum (arrowhead) and extensive subcutaneous emphysema (asterisk). The patient had a Brixia score of 14 at ER, 1 day of NIV prior to intubation and a SOFA score of 11 at ICU admission. The patient died during ICU hospitalization.

As reported in Table 1, we observed 25 cases of pneumomediastinum (65.79%), 24 cases of pneumothorax (63.16%) and 24 of subcutaneous emphysema (63.16%). Among 38 patients in Group 1, 14 subjects developed two manifestations sites of barotrauma (36.84%), 10 presented simultaneously pneumothorax, pneumomediastinum and subcutaneous emphysema (Figure 1) (26.32%), and 6 cases of pneumothorax were bilateral (6/24, 25%).

### 3.2. Clinical and Ventilatory Parameters

As shown in Table 2, patients who developed barotrauma are younger compared to Group 2 patients (mean 56.32 years vs. 61.63 years). The values of the statistical indices related to body mass index (BMI) were similar in both groups (mean 28.41 kg/m^2^ vs. 29.40 kg/m^2^); all included patients were generally overweight or slightly obese (BMI range 25–35 kg/m^2^) at the time of admission.

The mean values of the SOFA score were higher in patients with barotrauma (mean value 7.08) compared to the non-barotrauma group (mean value 5.93), while the values of the SAPS II score result was similar in the two groups. There is also a significant difference in the number of NIV days in prior intubation in the two study groups, being higher in the barotrauma compared to non-barotrauma patients (mean 4.20 vs. 2.75 days).

The PaO_2_/FiO_2_ ratio is significantly below the minimum threshold, indicating severe respiratory failure (defined with values < 100 mmHg), both at ICU admission and at the beginning of MV. However, no significant differences were noted among the two groups, as they were similar for PEEP values during NIV and at the beginning of MV.

Concerning mortality, rates were similar in the two groups: among the patients with barotrauma, 12/38 died (mortality rate of 31.58%), whereas in the group of patients with no barotrauma 28/79 died (mortality rate of 35.44%).

### 3.3. Brixia Score

**Figure 2:**
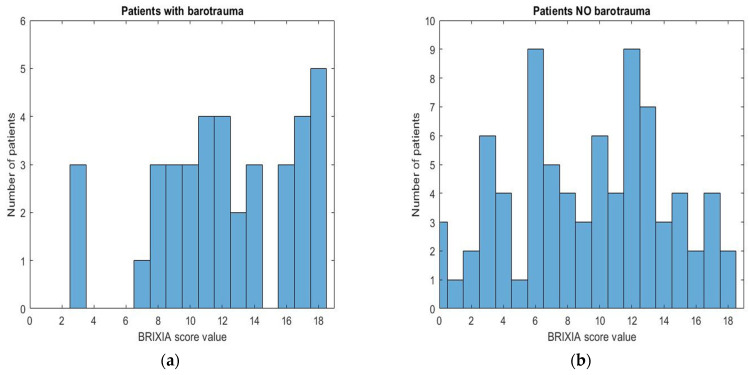
Graphic representation of the Brixia score (BS) value distribution in the two study groups: (**a**) Group 1—barotrauma, (**b**) Group 2—No barotrauma.

A preliminary analysis was performed assessing the distribution of BS values for both sample groups. Plotting the BS data of the Brixia scores, the histograms shown in Figure 2 were obtained: among patients with barotrauma the BS values are higher than those recorded patients without barotrauma (mean 12.18 vs. 9.28). Statistical indices regarding the distribution of the BS are summarized in Table 3.

**Figure 3:**
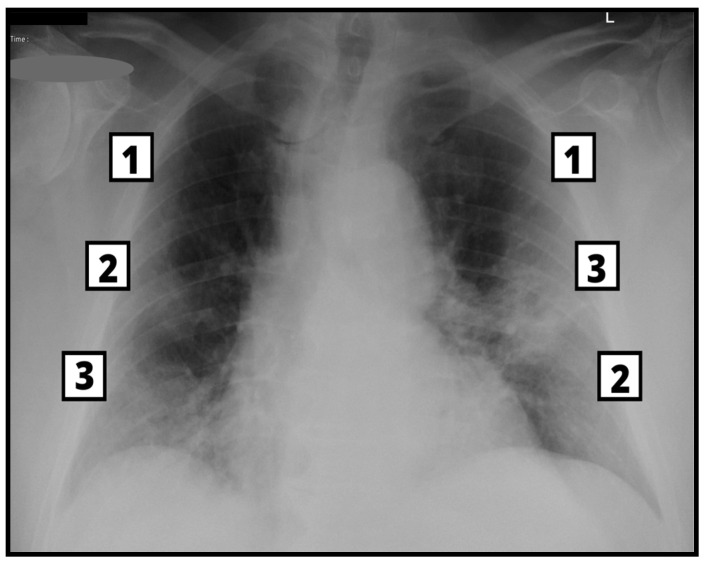
Frontal CXR in a 75-year-old male with SARS-CoV-2 infection, with a calculated Brixia score of 12. Days of NIV were 4 and SOFA score at ICU admission 12. The patient did not develop barotrauma and survived ICU hospitalization and acute infection.

**Figure 4:**
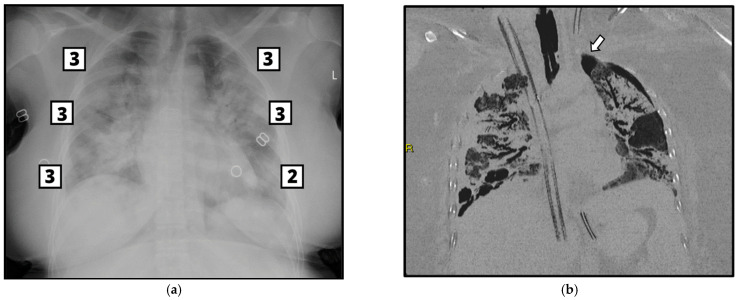
(**a**) CXR of a 37-year-old woman with severe bilateral COVID-19 pneumonia (Brixia score 17; days of NIV 0; SOFA score 5). The patient was directly intubated at ER admission and after 20 days of mechanical ventilation developed left-side pneumothorax, as shown on chest CT with coronal reconstruction (arrow, (**b**)). The patient was alive at the end of the study period.

Mann–Whitney U test, used with significance *p* < 0.05, allows us to state that between the two study groups there is a significant statistical difference concerning the values of the BS, which are significantly higher in patients with barotrauma (Table 3, Figure 3 and Figure 4).

### 3.4. Logistic-Regression Modelling

Logistic-regression analyses were performed to obtain a mathematical model that could identify the risk of barotrauma. The expected model outcome is the ability to associate between the parameters collected, a variable, or a combination of variables that may be useful in quantitatively assessing the likelihood that the patient will or will not develop barotrauma.

Simple univariate logistic regression models have been developed and the results, with coefficients and the Akaike Information Criterion (AIC) value for the model obtained for each variable, are shown in Table 4. The penultimate column of this table highlights the *p*-values related to slopes less than 0.05 of the variables having an increasing impact as their values increase. These indicate that the variables BS, SOFA score, and NIV days are the only characteristics with a *p*-value less than 0.05 and with the positive sign of their respective coefficient as expected, i.e., the higher the regressor value, the higher the probability of developing barotrauma. Therefore, it can be inferred that these three variables influence the probability that the patient will or will not develop barotrauma.

A *p*-value reported in the column Pr (≥|z|) is returned that must be less than 0.05 (for a 95% confidence level). The intercept (i.e., the value of the logit obtained from the regression when the regressor value is 0) and the slope (i.e., the regressor coefficient) are tested to verify the rejection of the null hypothesis that their values are not significantly different from zero.

In multiple logistic regression, Brixia Score, SOFA score, and NIV days were used simultaneously as independent variables to identify an initial model. After the first model was obtained with all predictors, three more models were obtained using possible combinations of the three independent variables involved (Table 5). Among the four models, the one showing the lowest AIC value is the first one, which uses the three independent variables simultaneously.

As per Table 4, the *p*-value reported in the column Pr (≥|z|) is returned that must be less than 0.05 (for a 95% confidence level). In multiple logistic regression studies, as with simple logistic regression, the barotrauma variable was set as the response variable, which can take the value of 1, indicating the development of pulmonary barotrauma (success), and 0, no occurrence of barotrauma. AIC is the Akaike Information Criterion: the lower its value, the better the model.

## 4. Discussion

The retrospective study sample included 117 SARS-CoV-2 patients admitted first to the Emergency Department and then to the intensive care unit (ICU) for mechanical ventilation, in these patients we detected a high incidence of barotrauma (38/117, 32.5%). Although in our group of those studied with and without barotrauma the mortality rate was similar, the literature suggests an adverse impact of barotrauma on the clinical outcome of SARS-CoV-2 patients in terms of hospitalization, length of ICU stay, and hospital mortality [3,16,21,22,23,24]. Specifically, mortality rates were reported up to 44.6% as pneumomediastinum-associated [23], and barotrauma was also independently associated with mortality [24]. The association of mortality with the presence of barotrauma, however, was not deeply analyzed in the current paper, being beyond the main stated purposes.

Chest X-ray is one of the first investigations performed in the Emergency Room in patients with respiratory symptoms and risk of pneumonia; therefore, our aim was to evaluate whether radiographic findings at the time of ER admission could have been helpful in identifying those at risk of barotrauma during the first peak of the COVID-19 pandemic. For this purpose, we tested the Brixia score, a radiological score used for quantifying and monitoring SARS-CoV-2 disease progression [18].

In our analysis, the distribution of the Brixia score in the two groups are quite different; in fact, among patients with barotrauma the values of the Brixia scores are higher (mean value 12.18) compared to the non-barotrauma group (mean value 9.28). Similarly, the median and modality indices also indicate more severe CXR findings in the I group, in particular the modality equal to 18, which is the maximum Brixia score value. Based on these results, it is very likely that the Brixia score could help in identifying patients at risk of developing barotrauma. To corroborate this hypothesis, we conducted a simple logistic regression analysis. This also demonstrates the ability of the Brixia score to classify patients at risk of barotrauma. In the univariate regression analysis, the SOFA SCORE calculated at ICU admittance and the number of the days of NIV prior to intubation emerged as significant variables. Therefore, multivariate logistic regression has been tested to identify the best logistic regression model: the first model was obtained using the Brixia score, SOFA score and NIV days simultaneously; the other three models were acquired using possible combinations of these variables. All models were found to be effective, but the best predicting model for barotrauma was obtained using the simultaneous employment of these three independent variables (Brixia score, SOFA score and NIV days).

In this work, we also assessed the distribution of several clinical parameters in patients with (Group 1) and those without barotrauma (Group 2). Subjects of the Group 1 were younger than the patients without barotrauma, values of BMI in Group 1 and Group 2 were similar: both results agreed with the data in the literature [16,25,26]. The distribution of the SOFA score values were higher in barotrauma patients, as opposed to the SAPSII score values which were similar in the two datasets. As indicated above, SOFA score also resulted in one of the most significant variables selected using univariate regression, implying an association with the risk assessment of barotrauma.

Finally, we investigated the relationship between barotrauma and ventilatory settings. Some studies hypothesize that barotrauma in COVID-19 patients would be associated with the inflammatory response to SARS-CoV-2 infection, suggesting a less-important pathophysiological role of the ventilator parameters [22,27]. In this research, we recorded the PEEP values during NIV and at the beginning of mechanical ventilation and we could not demonstrate significant differences in the two groups of patients. A very interesting fact is the marked difference in the number of days spent on noninvasive ventilation between patients with and without barotrauma: the average number of days of NIV calculated for the records contained in the barotrauma dataset is 4.2 days, whereas the average value of days of NIV experienced by patients without barotrauma is 2.75. Therefore, it could be hypothesized that the number of days of NIV prior to intubation are one of risk factors in the development of barotrauma; however, due to the limited amount of data available, the result may be unreliable.

The present study has some limitations; first of all is the limited sample size and its retrospective nature. Moreover, due to poor clinical conditions, a significant number of patients (86/117, 73.50%) performed CXR in a single antero-posterior projection, which may have affected the quality of the images and the analysis of the Brixia score. Another important drawback is that the starting lung conditions before COVID were not available; this is due to the emergency situation of the first pandemic peak and that no previous CT or X ray studies prior to COVID infection were available. Finally, other clinical/ventilatory factors might influence the patients’ outcome; however, confounding factors cannot be completely controlled in a retrospective study and, specifically in this case, in a severe emergency contingency.

## 5. Conclusions

Three independent variables, both radiological (Brixia score on CXR at ER admission) and clinical (SOFA score and NIV days at ICU admission) showed a potential association with barotrauma in COVID-19 infection. As affidavit of the recent pandemic, the present results firstly highlight that pulmonary barotrauma was not infrequent, while also being a clinically relevant complication in mechanically ventilated COVID-19 patients during the first peak. Moreover, despite this, the scenario currently appears less probable, and proof of these correlations may practically help to further deepen the understanding the consequences of SARS-CoV-2 infection on the lungs and possibly to furnish a novel combined approach to assess this complication in other ARDS scenarios.

## Figures and Tables

**Table 1:** Number of cases and percentage of clinical manifestations of barotrauma (pneumomediastinum, pneumothorax, subcutaneous emphysema), in Group 1 (38 patients).

Type and Combination of Barotrauma	Number of Cases	Percentage (n = 38 pts)
Pneumomediastinum	25	65.79%
Pneumothorax	24	63.18%
Subcutaneous emphysema	24	63.18%
Pneumomediastinum and subcutaneous emphysema	9	23.68%
Pneumomediastinum and pneumothorax	5	13.16%
Pneumothorax and subcutaneous emphysema	1	2.63%
Pneumothorax, pneumomediastinum and subcutaneous emphysema	10	26.38%

**Table 2:** Values of median, interquartile range (IQR), mean, and standard deviation of the common variables in both groups of patients (Group 1—barotrauma, Group 2—no barotrauma).

	Barotrauma Dataset				No Barotrauma Dataset			
	Median	IQR	Mean	Std Dev	Median	IQR	Mean	Std Dev
**Age**	57.00	15.50	56.32	13.17	62.50	12.50	61.63	9.83
**BMI**	27.34	5.62	28.41	5.41	27.77	6.35	29.40	5.09
**SOFA score (0–24)**	7.00	4.00	7.08	2.71	6.00	3.50	5.93	2.27
**SAPS II (0–163)**	37.00	16.00	38.35	13.76	39.00	10.00	39.93	10.71
**BRIXIA score (0–18)**	12.00	7.00	12.05	4.33	10.00	7.00	9.38	4.83
**P\F at ICU admission**	103.00	47.00	112.84	48.29	100.00	49.25	117.75	52.59
**P\F at MV beginning**	65.50	37.00	76.57	26.75	73.00	35.50	79.21	26.11
**PEEP at MV beginning**	15.00	8.00	14.56	3.71	15.00	4.00	15.16	3.06
**Mean PEEP before MV**	15.00	4.00	14.21	2.69	14.50	4.38	14.30	2.93
**Maximum PEEP before MV**	16.00	3.00	16.15	3.03	16.00	4.00	15.72	3.23
**Days in NIV before MV**	4.00	3.75	4.20	3.19	2.00	3.00	2.75	2.17
**Integral of the PEEP before MV**	53.85	53.00	57.12	39.74	32.50	38.56	39.52	33.23

BMI: body mass index. SOFA score: sequential organ failure assessment score. SAPS II score: simplified acute physiology score II. P/F: arterial partial pressure of oxygen [PaO_2_]/fraction of inspired oxygen [FiO_2_]. ICU: intensive care unit. MV: mechanical ventilation. PEEP: positive-end-expiratory pressure. NIV: non-invasive-ventilation.

**Table 3:** Statistical indexes of the Brixia score (BS) in the two study groups and results of Mann–Whitney U test (significance *p* < 0.05).

Brixia Score at CXR	Barotrauma Data Set (Group 1, n = 38 pts)	NO Barotrauma Data Set (Group 2, n = 79 pts)
Mean	12.18	9.28
Median	12	10
Standard deviation	4.35	4.78
Interval	15	18
Minimum value	3	0
Maximum value	18	18
	**Mann–Whitney U-test on Brixia score median**	
	Z-value	−2.67
	Critical z	±1.96
	Decision rule	z ≥ |1.96|
	Result	**H0 rejected**

CXR: chest X-ray.

**Table 4:** Coefficients of the simple logistic regression models of the eight independent variables.

Predictors	Coefficients	Estimate	Std. Err.	Z Value	Pr (>|z|)
**Age**	Intercept Slope	1.76 **−0.04**	1.100 0.019	1.602 −2.316	0.1092 0.0206
**BMI**	Intercept Slope	0.31 **−0.04**	1.311 0.045	0.233 −0.881	0.8160 0.3780
**N. comorbidities**	Intercept Slope	−0.58 **−0.20**	0.280 0.208	−2.057 −0.954	0.0397 0.3402
**Brixia Score**	Intercept Slope	−2.13 **0.13**	0.560 0.046	−3.794 2.723	0.0001 0.0065
**SOFA score**	Intercept Slope	−2.01 **0.19**	0.589 0.083	−3.414 2.296	0.0006 0.0217
**SAPS II score**	Intercept Slope	−0.30 **−0.01**	0.714 0.018	−0.426 −0.676	0.6700 0.4990
**NIV days**	Intercept Slope	−1.45 **0.13**	0.340 0.056	−4.249 2.409	2.14e-05 0.0160
**Mean PEEP**	Intercept Slope	−0.72 **−0.01**	1.059 0.073	−0.675 −0.152	0.4990 0.8800

**Table 5:** Estimates of the coefficients obtained from multiple logistic regression of the four combinations of predictors (Brixia score, SOFA score, NIV days).

Model	Predictors	Coefficients	E Stimate	Std. Err.	z Value	Pr (>|z|)	AIC	AIC c
1	Brixia score SOFA score NIV days	Intercept Brixia score SOFA score NIV days	−4.15 0.12 0.19 0.17	1.087 0.057 0.098 0.077	−3.821 2.073 1.910 2.161	0.0001 0.0381 0.0562 0.0307	99.91	100.40
2	Brixia score NIV days	Intercept Brixia score NIV days	−2.95 0.12 0.15	0.835 0.056 0.073	−3.530 2.240 2.073	0.0004 0.0251 0.0382	101.73	102.02
3	Brixia score SOFA score	Intercept Brixia score SOFA score	−3.17 0.11 0.17	0.908 0.054 0.095	−3.487 1.982 1.835	0.0005 0.0475 0.0664	103.73	104.02
4	SOFA score NIV days	Intercept SOFA score NIV days	−2.88 0.21 0.14	0.819 0.097 0.071	−3.515 2.107 2.013	0.0004 0.0351 0.0441	102.64	102.93

## Data Availability

The collected and analyzed data of the current study are not publicly available due to individual privacy policy. However, clarifications are available from the corresponding author upon reasonable request.
